# Human disturbance increases coronavirus prevalence in bats

**DOI:** 10.1126/sciadv.add0688

**Published:** 2023-03-31

**Authors:** Vera M. Warmuth, Dirk Metzler, Veronica Zamora-Gutierrez

**Affiliations:** ^1^ Division of Evolutionary Biology, Faculty of Biology, Ludwig-Maximilians-Universität München, Großhaderner Straße 2, 82152 Martinsried, Germany.; ^2^ CONACYT - Centro Interdisciplinario de Investigación para el Desarrollo Integral Regional Unidad Durango (CIIDIR), Instituto Politécnico Nacional, Durango, México.

## Abstract

Human land modification is a known driver of animal-to-human transmission of infectious agents (zoonotic spillover). Infection prevalence in the reservoir is a key predictor of spillover, but landscape-level associations between the intensity of land modification and infection rates in wildlife remain largely untested. Bat-borne coronaviruses have caused three major disease outbreaks in humans: severe acute respiratory syndrome (SARS), Middle East respiratory syndrome, and coronavirus disease 2019 (COVID-19). We statistically link high-resolution land modification data with bat coronavirus surveillance records and show that coronavirus prevalence significantly increases with the intensity of human impact across all climates and levels of background biodiversity. The most significant contributors to the overall human impact are agriculture, deforestation, and mining. Regions of high predicted bat coronavirus prevalence coincide with global disease hotspots, suggesting that infection prevalence in wildlife may be an important factor underlying links between human land modification and zoonotic disease emergence.

## Teaser

Bats in human-disturbed landscapes have higher coronavirus infection rates than bats in less disturbed habitats.

## INTRODUCTION

There is now little doubt that human land modification is a major driver of infectious diseases in humans that have an origin in animals (“zoonoses”) (*1*–*3*). This means that animal-to-human transmissions of infectious agents (“zoonotic spillover”) occur more frequently in human-modified habitats than in less disturbed areas. At the most fundamental level, spillover is a function of pathogen prevalence in the reservoir, contact rate between reservoir, and recipient host and the likelihood of infection after contact (*4*). A recent study could show that animal species capable of harboring zoonotic agents are more abundant in human-modified versus less modified habitats, suggesting that land modification increases contact rates between humans and zoonotic hosts (*5*).

Land modification has also been suggested to increase the likelihood and severity of infection in wildlife (*3*). In particular, it is thought that the degradation and fragmentation of habitats that often accompany human land modification expose animals to prolonged stress as they adapt to the loss or redistribution of resources (e.g., food, sleeping/roosting sites, and mates) (*3*). The immunosuppressive effects of chronic stress in mammals are well known (*6*), and there is increasing evidence for human disturbance causing physiological stress in wild animals (*7*–*10*), yet few studies have robustly linked anthropogenic stressors with infection prevalence in wildlife across large spatial scales (*3*).

In the past 20 years, zoonotic coronaviruses have caused three major disease outbreaks in humans: severe acute respiratory syndrome (SARS), Middle East respiratory syndrome, and coronavirus disease 2019 (COVID-19) (*11*, *12*). All three of these outbreaks started with spillover from bats to other animals/humans (*12*), making the Coronaviridae a family of considerable zoonotic concern. As the evolutionary reservoir of α and β coronaviruses (*13*), bats (order Chiroptera) play an important role in the ecology of coronavirus spillover (*14*). Understanding how human land modification influences coronavirus infection dynamics in this important reservoir is consequently vital to managing spillover risk.

Here, we investigate the effect of human land modification on the prevalence of coronavirus infection in bats by statistically linking high-resolution spatial datasets of global anthropogenic stressors (*15*) with coronavirus infection data from worldwide bats extracted from the scientific literature.

## RESULTS

**Fig. 1.**
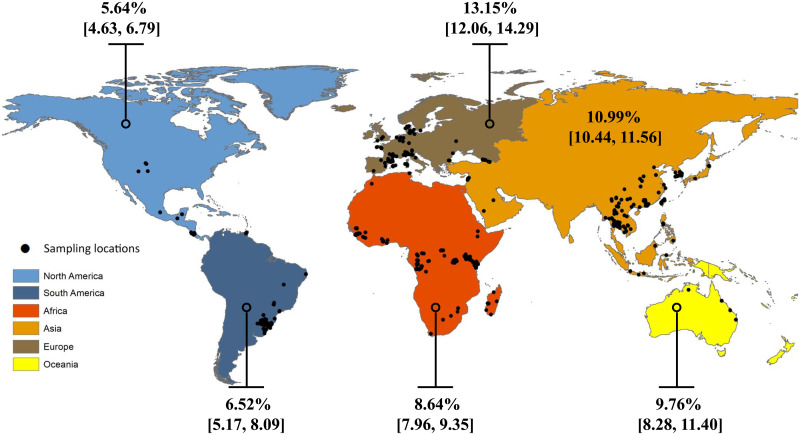
Geographic origin of coronavirus surveillance data included in our dataset. Observed prevalence (±95% CI) in wild bats averaged over continents. Raw data are in table S2.

Of the 151 studies retained for full-text review, only 74 studies met our eligibility criteria. The most common reason for exclusion was a lack of information on the geographic origin of samples (*n* = 37), followed by pooling of data from individuals sampled over a large geographic area (>50 km, *n* = 16) and pooling of material from more than one individual (*n* = 15) (table S1). Our final dataset contains coronavirus infection/noninfection data for 26,723 bats from 309 species, representing 15 families and 111 genera (table S2). Observed coronavirus prevalence shows considerable geographical variation, ranging between 0 and 100% [global mean: 9.61%; 95% confidence interval (CI): 2.25, 59.78] among the *n* = 432 sample locations included in our dataset (Fig. 1 and fig. S1). Extensive variation, both across space and time, is common in viral diseases and is likely due to the large number of factors influencing their occurrence (*16*–*18*). To test the hypothesis that human land modification (human impact hereafter) is a significant factor underlying the spatial patterns that we observe, we modeled the relationship between infection presence and prevalence and quantitative estimates of global human land modification using zero-inflated mixed-effects logistic regression models (*19*, *20*). We used the contemporary human land modification dataset by Theobald *et al*. (*15*). This dataset provides estimates of the degree of human modification (*H*) for ~2017 and includes 14 anthropogenic stressors from five categories (Supplementary Text and fig. S2).

**Fig. 2.**
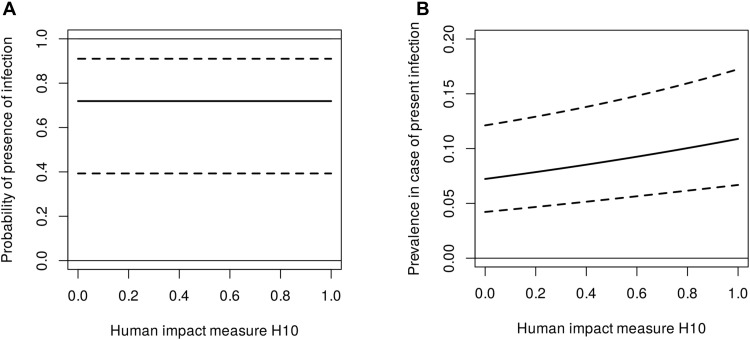
Global predicted effect of overall human impact H10. Predicted probability of coronavirus presence (**A**) and (**B**) prevalence (conditioned on presence) for an average locality. Dashed lines show 95% confidence ranges. Note that human impact was modeled at a spatial resolution of 10 km^2^.

We first tested the effect of overall human impact (fig. S2A) at both 10-km^2^ (H10) and 50-km^2^ (H50) resolution. After accounting for all confounders (see Materials and Methods), H10 showed a significant effect on coronavirus prevalence (Wald test, *P* = 0.00078), but not presence (Wald test, *P* = 0.43; Table 1). At the global level, i.e., at an average sampling locality, the predicted probability of coronavirus presence in wild bats is high (mean, 0.72; SE, 0.14; Fig. 2A), and prevalence increases with the intensity of human impact (Fig. 2B). Very similar results were obtained for analyses using H50 instead of H10 (table S3).

**Table 1.** Model coefficients for the zero-inflated binomial logistic regression generalized linear mixed model. Shown are the results for human impact at 10-km spatial resolution (H10). Coefficients for the same model but using human impact at 50-km spatial resolution are presented in table S3. Note that, for the zero-inflation models, larger values increase the probability of the absence of infection.

Conditional model fixed effects	Estimate	*P* value (Wald test)
Intercept	−2.67	1.93 × 10^−07^
H10	0.449	0.00078
Richness	−0.00047	0.86
Absolute value of latitude	0.00779	0.49
		
**Conditional model random effects**	SD	
Climate	0.370	
Species	0.967	
Study	1.06	
**Zero-inflation model fixed effects**	Estimate	*P* value (Wald test)
Intercept	−0.776	0.428
Richness	−0.00026	0.965
Absolute value of latitude	−0.00665	0.75
**Zero-inflation random effects**	SD	
Climate	0.297	
Species	0.617	
Genus	0.356	
Study	1.191	

**Fig. 3.**
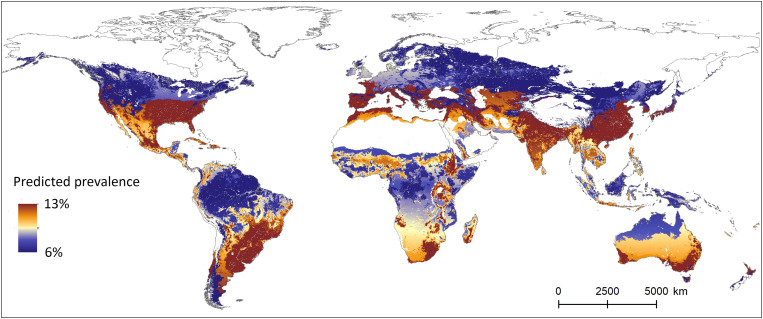
Predicted coronavirus prevalence in worldwide bats. Heatmap of predicted coronavirus prevalence conditioned on virus presence. Areas with insufficient data for extrapolation are indicated in white.

Visualization of the model predictions for coronavirus prevalence shows global regions where coronavirus spillover from bats is most likely to occur (Fig. 3 and fig. S3). Some of the coronavirus prevalence hotspots predicted by our model, notably Western Europe, East Asia, and India, closely coincide with previously identified hotspots of zoonotic disease emergence in humans, which have also been unambiguously linked to human-induced drivers (*21*–*23*). The spatial overlap of global disease hotspots and bat coronavirus prevalence hotspots in regions under substantial human pressure suggests that increased infection prevalence in wild animals may be a central outcome of human land modification and an important factor underlying links between human disturbance and zoonotic disease emergence (*3*).

The 14 stressors contributing to the overall measure of human impact can be grouped into five major categories (*15*): agriculture and deforestation (“Ag”), intrusions and pollution (“In”), transportation (“Tr”), energy production (“En”), and urban and built-up (“Bu”) (fig. S2, A to F). To test which stressor(s) have the biggest effect on bat coronavirus prevalence, we fitted five additional models to our data. In each of these models, we replaced H10 as explanatory variable for conditional prevalence by one of these five stressor types at a 10-km^2^ spatial resolution. After multiple-testing correction, only Ag10 and En10 had significant positive effects on conditional prevalence (Wald test, Bonferroni-Holm corrected *P* < 0.0002 for both Ag10 and En10). Additional testing confirmed that these two stressor types drive the effect of overall human impact (see Materials and Methods).

**Fig. 4.**
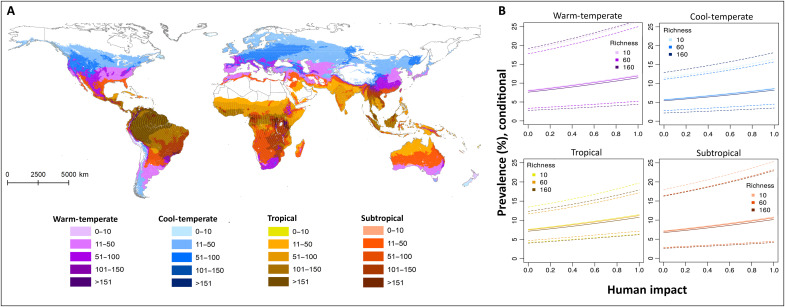
The combined effects of human impact, climate, and mammal species richness on coronavirus prevalence in bats. (**A**) Geographical distribution of mammal species richness (color gradients) in warm-temperate (purple), cool-temperate (blue), tropical (yellow-brown), and subtropical (red) climates. (**B**) Predicted relationship between human impact and coronavirus prevalence (%) for average bat species in warm-temperate (top left), cool-temperate (top right), tropical (bottom left), and subtropical (bottom right) climates, and for low (10; light color), intermediate (60; intermediate color), and high (160; dark color) mammal species richness. Dashed lines show 95% confidence ranges. Note that predicted prevalence is conditioned on virus presence.

Human land modification often interacts with climate and/or biodiversity in its effect on pathogen transmission and zoonotic disease emergence (*22*, *24*, *25*). We explored the predicted effect of human impact on bat coronavirus prevalence for the different climate regions and levels of mammal species richness in our dataset (Fig. 4A and fig. S4). We find that higher mammal species richness is associated with lower coronavirus prevalence in all climate regions and across all levels of human impact (Fig. 4B and fig. S5); however, we note that this effect is not strong enough to draw definitive conclusions about a potentially beneficial role of high background mammal richness on coronavirus prevalence in bats.

**Fig. 5.**
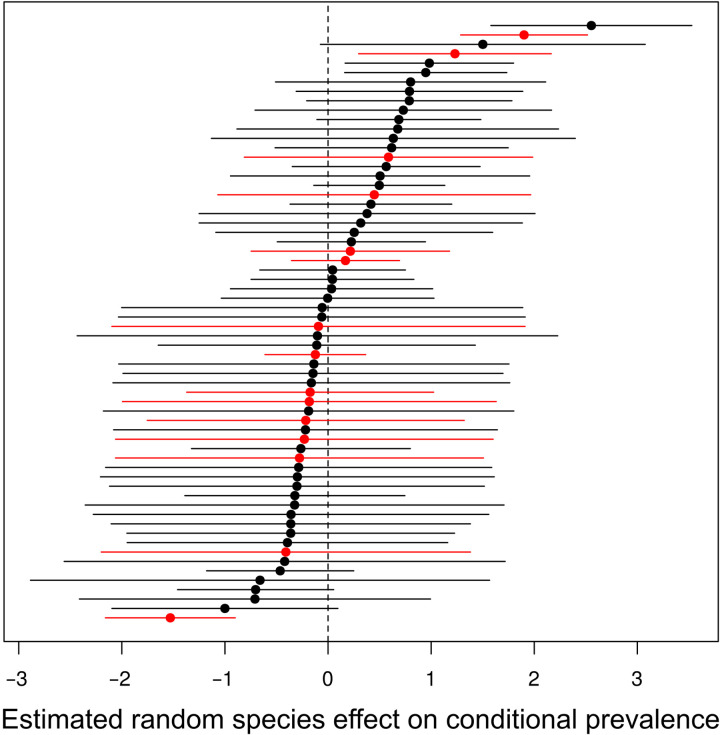
Estimated random host species effects on conditional prevalence (±2 SD) for East and Southeast Asian bat species. Bat species whose ranges are restricted to East and Southeast Asia and for which coronavirus presence/absence data from at least 10 individuals was available. The larger the values, the higher the fraction of infected individuals in a given species if there is an infection in the population. Negative values mean that even if virus is present in the population, it tends to affect only few individuals. Species belonging to the family Rhinolophidae are in red. See table S4 for a list of the species, ordered by effect size, included in this figure.

The best-fitting model, which allows for a random effect of host species, explained the data significantly better than a model in which this species-level effect was replaced by a genus-level effect (analysis of deviance, *P* < 2.2 ×10^−16^). Tests for residual phylogenetic signals at higher taxonomic levels were not significant. As the preferred bat hosts for SARS-like coronaviruses (*26*) and the likely reservoir for the SARS–coronavirus 2 (CoV-2) progenitor (*27*), the family Rhinolophidae (horseshoe bats) warrants particular attention. Focusing on South and Southeast Asia as the current diversity hotspot for rhinolophid hosts of SARS-like coronaviruses (subgenus *Sarbecovirus*) (*28*), we asked whether the Rhinolophidae have higher rates of coronavirus infection than bats from other codistributed families. We compared predicted coronavirus prevalence among bat species in our dataset whose ranges are restricted to South and Southeast Asia (table S4) and find no consistent pattern of elevated coronavirus prevalence in the family Rhinolophidae (Fig. 5, Rhinolophidae in red). While some rhinolophid species had relatively high predicted prevalence, the two species considered the most likely sources of the SARS-CoV-2 progenitor—*Rhinolophus pusillus* and *Rhinolophus affinis* (*27*, *29*, *30*)—ranked 46 and 26, respectively, among the 64 species included in this analysis (table S4). Predicted prevalence was also comparatively low for *Rhinolophus malayanus* (rank 57; table S4), another rhinolophid species that has been proposed as a potential reservoir of the SARS-CoV-2 progenitor (*29*).

## DISCUSSION

Our findings show that land modification increases infection prevalence in the global bat population and that this effect appears to be driven by activities that reduce available bat habitats, food sources, and roosting spaces. In a recent review, deforestation and agriculture were identified as the top two most important anthropogenic threats to global bat diversity, each directly affecting more than 50% of threatened bat species (*31*). Globally, forests are the most important habitats for bats. The large effect of stressors in the category agriculture and biological harvesting of forests (Ag) likely reflects the loss of important forest habitats either directly through logging or through conversion to agricultural lands. Agricultural practices such as the use of insecticides or pest-resistant crop varieties additionally reduce foraging resources for insectivorous bats (*32*). The significant effect of stressors in the category energy production and mining (En) may reflect the destruction of both above-ground foraging habitats and subterranean roosting places as a consequence of mining activities and other means of energy production including wind turbines. Wind turbines not only cause high rates of mortality (*33*) but also further reduce available foraging habitat for bats shown to actively avoid foraging near them (*34*).

The destruction and/or reduction of key resources may expose bats to physiological stress (*3*). The immunosuppressive effects of chronic stress are well known (*6*), and mounting evidence shows that human disturbance is a source of chronic stress in wild animals (*7*, *10*, *35*). In bats, stress, including ecological stress, has been linked to (re)activation of latent viruses and increased viral shedding (*36*–*38*). By revealing a significant association between anthropogenic stressors and pathogen prevalence in a major viral reservoir, our results suggest that ecological stress is a major factor linking human land modification and zoonotic disease emergence.

We found no evidence for consistently higher coronavirus prevalence in horseshoe bats (family Rhinolophidae) compared to other bat families. In particular, the three species that are thought to be the most likely reservoirs for the progenitor of SARS-CoV-2, which crossed into the human population on more than one occasion (*11*), have comparably low predicted prevalence (table S4). This result is consistent with the idea that additional mechanisms not addressed in our study determine the probability of spillover alongside pathogen prevalence in donor hosts—bat or otherwise (*39*). Central among these will be mechanisms that increase the rate of contact between donor and recipient hosts (*4*). Wildlife wet markets likely played a central role in the outbreaks of both SARS (2002–2004) and COVID-19 (*40*, *41*). The trading of animals in general and wet markets in particular brings species that would otherwise hardly meet into close contact with one another and with humans. Already 10 years ago, the global wildlife trade involved up to one billion direct and indirect contacts among wildlife, humans, and domestic animals (*42*), and increasing evidence suggests a strong link between wildlife trade and zoonotic disease risks (*43*).

Overall, our analysis likely provides rather conservative estimates of coronavirus prevalence in wild bats because molecular assays designed to target specific lineages or strains (e.g., SARS-related coronaviruses; subgenus *Sarbecovirus*) might have a lower sensitivity to detect more distantly related species in this large and diverse family. Conversely, pan-coronavirus assays, another frequently used type of assay, may have overall lowered sensitivity. On the other hand, two sources of bias could result in an overestimation of prevalence: (i) A publication bias toward studies reporting high coronavirus prevalence could lead to an overestimation of overall coronavirus prevalence. The influence of this type of bias is likely minor, as 68 of the 74 studies included in our analysis (92%) report infection data from multiple species, localities, and/or time points. Hence, these datasets are characterized by a predominance of low or zero prevalence values and are therefore unlikely to be biased toward high prevalence values (table S2). (ii) A publication bias toward studies explicitly investigating and finding a significant effect of human land modification on coronavirus infection in bats could potentially lead to an overestimation of the effect of human impact on prevalence. This form of publication bias is also unlikely, as the 74 studies contributing data to our analyses address a diverse range of research questions with only a single study (study ID 26; table S1) explicitly investigating the influence of human disturbance on coronavirus infection in bats (and finding no effect).

Our predictions (Fig. 3 and fig. S3) take into account local values for climate, mammalian species richness, latitude, and human impact, but not other geographic characteristics or the composition of the local bat fauna. Bat species occurring in areas of high predicted prevalence may have adapted to local conditions, including the factors that we model explicitly, and be less susceptible to coronavirus infection than predicted. Fitting a model that takes into account the composition of the local bat fauna or adaptation to local conditions was not reasonably possible with the available data, given that our simpler model already returns predicted prevalence estimates with relatively broad CIs. These uncertainties notwithstanding the effect of human impact on coronavirus prevalence in bats are significant, suggesting that land modification drives zoonotic disease emergence through at least two mechanisms: by increasing infection prevalence in the reservoir (this study) and by increasing contact between the reservoir and recipient hosts, human, or other (*5*).

To predict and mitigate spillover risk of potential zoonotic pathogens, our findings emphasize the necessity to monitor not only their presence but also their prevalence in wildlife populations. Our analyses reveal that hotspots of coronavirus prevalence coincide with regions under intense human pressure, suggesting that prevalence data may become increasingly important as human impact expands. Our results also identify a handful of regions, notably including the Eastern United States and India, where increased surveillance efforts may be especially critical.

## MATERIALS AND METHODS

### Experimental design

In the present study, we investigate the effect of human land modification on coronavirus presence and prevalence in bats. To do so, we statistically link high-resolution spatial datasets of anthropogenic stressors (both individual stressor categories and their cumulative effect) with global coronavirus surveillance records using zero-inflated mixed-effects logistic regression models (*19*, *20*).

### Literature review

We identified potentially relevant studies from Web of Science with the topic search terms defined as ((coronavirus*) AND (prevalence*) AND (bat* OR Chiroptera)). This literature search was first conducted in May 2020 and repeated in September 2022. In September 2022, we additionally checked the database of coronavirus (sero)prevalence compiled by Cohen *et al.* (*44*) for studies that we may have missed. We screened through the title and abstract of all the resulting candidate studies (*n* = 340) and selected for full-text review only those that seemed likely to contain quantitative information on the prevalence of coronavirus RNA in samples collected from wild bats (*n* = 151; table S1).

To be eligible for inclusion, the 151 candidate articles were required to comply with the following inclusion criteria. First, we considered only studies that used polymerase chain reaction–based methods for the detection of coronavirus RNA in wild-caught bats; studies reporting coronavirus seroprevalence and seroprevalence records within a study that reported both types of data were excluded. Second, we required studies to contain information on the location of sampling sites in sufficient detail to allow geo-referencing at a spatial resolution of ≤50 km^2^. If no GPS coordinates were provided by the authors, but sampling localities were identifiable based on place names, then we used the website www.asturnatura.com/sinflac/calculadora-conversiones-coordenadas.php to retrieve the geographical coordinates of sampling localities. In some instances, no geographical coordinates or identifiable place names were given, but sampling localities were instead visualized on regional-scale (∼50 to 200 km) maps; in these cases, we inferred their geographical coordinates through side-by-side comparison with the same area on Google Maps. Third, we only included coronavirus presence/absence data generated from feces, fecal swabs, anal swabs, or alimentary tract tissue; data generated from urine samples or oral swabs were excluded, as detection probability of coronavirus RNA from these materials is relatively lower (*45*). We also excluded coronavirus presence/absence data generated from pools of fecal samples; of coronavirus presence/absence or prevalence data calculated from samples of multiple species or anonymous species; and of presence/absence or prevalence data calculated from individuals of the same species but sampled over a large area (>50 km). We further excluded studies screening for coronavirus nucleic acids in bats using a metagenomics/metatranscriptomic approach as well as reviews, editorials, and other articles related to bats and coronaviruses but which did not present primary data on coronavirus presence/prevalence in wild bats. Of the 151 studies subjected to full-text revision (table S1), 74 met our selection criteria and were retained for data extraction (table S2). In our database, each data point represents coronavirus infection data (i.e., the fraction of coronavirus-positive bats) for individuals from the same species, sampled at a specific location and at a specific time.

### Statistical analysis

#### Observed prevalence

Observed prevalence summarized by continent (Fig. 1) and country (fig. S1) was calculated using the epiR package in R v. 4.0.3 (*46*).

#### Model fitting

To assess the effect of human land modification on the presence and prevalence of bat coronaviruses, we fitted zero-inflated binomial logistic regression generalized linear mixed models with the R package glmmTMB (v.1.1.4, TMB package v. 1.9.1, R v. 4.2.1) (*19*, *46*, *47*). Quantitative estimates of the intensity of human land modification for ~2017 were obtained from (*15*). We tested both the overall human impact, which is calculated as the cumulative effect of 14 anthropogenic stressors in the original study, and the effects of stressors from five major categories: Bu, Ag, En, Tr, and In (fig. S2, A to F). The following factors have been shown elsewhere to influence infectious disease dynamics at large spatial scales: climate (*24*, *48*), biodiversity (*23*, *49*–*51*), absolute latitude (*52*), and host phylogeny (*53*, *54*). We included climate (*55*), mammal richness (as a proxy for biodiversity; fig. S4A) (*56*), absolute latitude, and study ID into the models for both the zero-inflation probability (i.e., the probability of virus presence) and the conditional model (i.e., virus prevalence conditional on presence) to account for their potential confounding effects. Two versions of the climate data were considered: the original dataset (*55*), in which climate regions are defined on the basis of both temperature and moisture regimes (figs. S4B and S5 and table S5), and one where climate regions were defined by temperature regime only (Fig. 4, Table 1, and table S6).

In a preliminary model, we allowed for an additional random genus effect on prevalence (conditioned on presence), but we removed it for the final model as the estimated effects were negligibly small and led to convergence problems in some variants of our models. We allowed for possible random genus-level effects on presence, that is, on the zero-inflation component of the model. The categorical variables climate, host genus, host species, and study ID were also modeled as random effects. All factors were retained in the fitted model to correct for their expected confounding effects.

For each data point, we interpret the zero-inflation probability of the fitted model as the probability that the virus is absent in the population, and the fitted parameter p of the conditional binomial distribution corresponds to virus prevalence in our application. Note that a zero can appear in the data if a population is uninfected or if the virus is present in the populations but, by chance, only uninfected individuals were sampled. As CIs, we give two SE ranges on the linear (link) scale, transformed to conditional prevalence according to the logistic link function of the model. We calculated quantile residuals for our fitted models conditioned on the inferred random effects using the R package DHARMa v. 0.4.6 (*57*). With quantile plots and bootstrapped outlier tests, we checked the distribution assumptions for the quantiles and carried out Kruskal-Wallis tests to check whether the residuals show any signal of continent, country, or the feeding guild of the bat species. As we find no significant evidence of additional effects of countries or continents, it seems plausible that our predictions apply, to some extent, also to geographic regions from which we had no or few data points as long as these regions are not systematically different from the sampled region with respect to the other factors.

#### Significance test

We assessed the significance of human impact at a ~10-km^2^ grid-cell resolution, first, by Wald tests of the inferred model coefficient (as reported in R by the summary command for a model fitted with glmmTMB) and, second, by a simulation-based likelihood ratio test according to a parametric bootstrap approach. For the latter, we fitted a null model without H10 (but with all other variables in) to the data and simulated 1000 datasets according to the fitted null model. Then, we refitted the null model and our alternative model to each of the datasets and calculated the likelihood ratio. The simulation-based *P* value is then (*k* + 1)/1001, where *k* is the number of simulated datasets in which the likelihood ratio was at least as large as the original data.

#### Model selection

We set up two models, model A, in which human impact has an effect on both virus presence (i.e., the zero-inflation component of the model) and prevalence (i.e., the conditional component of the model), and model B, in which human impact has an effect on virus prevalence, but not presence. The quantile residuals of models A and B did not show any significant violations of model assumptions. Model A fitted the data significantly better than a null model without any effects of human impact (parametric bootstrapping test, *P* = 0.007). Model B also explained the data significantly better than the null model (parametric bootstrapping test, *P* = 0.005), and there was no significant difference between models A and B (parametric bootstrapping test, *P* = 0.31); however, model B had the better Akaike information criterion value (model B, 3446.24; model A, 3447.24; null model, 3455.40). We here report model outcomes only for model B.

In model B, human impact showed a significant effect on prevalence (i.e., the conditional model; Wald test, *P* = 0.00078), but not on the zero-inflation model, which describes the probability of the absence of the virus in a local population (Wald test, *P* = 0.43; Table 1). The residual diagnostics calculated with DHARMa did not show any significant violations of model assumptions (bootstrapped outlier test, *P* = 0.52). We found no evidence for associations of the quantile residuals with factors that we did not account for in our model (Kruskal-Wallis test; *P* = 0.42 for continent, *P* = 0.97 for country, and *P* = 0.55 for feeding guild). To check for possible effects on higher taxonomic levels, we carried out a phylogenetic contrasts analysis (*58*) for the residuals of models A and B. We applied the program contrast from the software package phylip v. 3.697 (*58*–*60*) through the interface provided by the R package Rphylip v. 0.1-23 (*61*) to compare intraspecies variation in the residuals to phylogenetic variation. For this analysis, we used the bat phylogeny inferred by (*62*) and pruned to the 259 species that were in our dataset using the R package ape v. 5.6-2 (*63*). The within-species variance was estimated 0.0841 for model A and 0.0832 for model B, and, in both cases, the phylogenetic between-species component estimation was only 7 × 10^−6^ or 3 × 10^−6^, respectively, and not significant (*P* = 1; the program phylip/contrast reported slightly negative chi-square values of −0.044 and −0.1, presumably due to numerical imprecisions like rounding errors).

To identify the anthropogenic stressor types that have the greatest effect on bat coronavirus prevalence, we fitted five additional models to our data. In each of these models, we replaced H10 as explanatory variable for conditional prevalence by one of five major stressor groups: Ag10 (agriculture and biological harvesting of forests), Bu10 (urban and built-up), En10 (energy production and mining), In10 (human intrusion, natural system modifications, and pollution), and Tr10 (transportations and service corridors), all at a 10-km^2^ resolution (fig. S2, A to E). To test whether the effects of Ag10 and En10 mask each other in a model that contains both variables, we set up a model that included both Ag10 and En10 (without H10). Last, we were interested to assess whether H10 as a cumulative measure has effects that are independent of the individual effects of Ag10 or En10. For this, we combined first Ag10, then En10, with H10 in a model to test whether the latter loses its significance when either of the former is included as separate variables.

After multiple-testing correction, only Ag10 and En10 had significant positive effects on conditional prevalence (Wald test, Bonferroni-Holm corrected *P* < 0.0002 for both Ag10 and En10). Additional testing confirmed that these two stressor types drive the overall effect of H10. In a model with both Ag10 and En10, the two effects did not mask each other, both variables were still significant (Wald test, *P* = 0.0026 for Ag10 and *P* = 0.0044 for En10). When we combined one of the two variables, Ag10 or En10, with H10 in a model, the effects of Ag10 and En10 were still significant (*P* = 0.0073 and *P* = 0.0027, respectively), whereas H10 lost its significance in both the model with Ag (*P* = 0.3000) and the model with En (*P* = 0.0530).
