# Supplementary material for: Human disturbance increases coronavirus prevalence in bats
Source: Sci Adv. 2023 Mar 31;9(13):eadd0688. doi: 10.1126/sciadv.add0688 (PMC10065436; doi:10.1126/sciadv.add0688)
Supplement: Supplementary file 1 — Supplementary Text Figs. S1 to S5 Legends for tables S1 and S2 Tables S3 to S6 [file sciadv.add0688_sm.pdf]

Science Advances
AAAS

Supplementary Materials for

Human disturbance increases coronavirus prevalence in bats

Vera M. Warmuth, Dirk Metzler, Veronica Zamora-Gutierrez

Corresponding author: Vera M. Warmuth, warmuth@bio.lmu.de; Veronica Zamora-Gutierrez, zamora.gtz@gmail.com

Sci. Adv. **9**, eadd0688 (2023)
DOI: 10.1126/sciadv.add0688

The PDF file includes:

Supplementary Text
Figs. S1 to S5
Legends for tables S1 and S2
Tables S3 to S6

Other Supplementary Material for this manuscript includes the following:

Tables S1 and S2

Supplementary Text

Spatial Variables

Human land modification

We used the measure of contemporary global human land modification (H) calculated by Theobald et al. (*15*), and available at dryad under the following link: https://datadryad.org/stash/dataset/doi:10.5061/dryad.n5tb2rbs1 (dataset 'gHMv1_1000m_2017_static_stressors.zip'; accessed 21/07/2020). For details of how H is calculated, please refer to (*15*). The measure of contemporary H as estimated by (*15*) (Fig. S2 A) includes 14 individual stressors from five categories: agriculture & harvesting of forests (Ag), urban and built-up (Bu), energy production & mining (En), transportation & service corridors (Tr), and human intrusions, natural system modifications & air pollution (In) (Fig. S2 B-F). The original dataset is at a spatial resolution of 1 km^2. To test whether the effect of human land modification on coronavirus prevalence differs depending on the spatial scale it is studied, we considered H at two spatial resolutions, 10 km^2 ('H10') and 50 km^2 ('H50'), respectively. The spatial resolution of the original dataset was changed by averaging across the values of the original cells.

Mammal species richness

We used an updated (2018) version of the total mammal species richness map originally derived from IUCN range maps (*56*) (source: https://biodiversitymapping.org/index.php/mammals/; accessed 21/07/2020). In this map, global mammal species richness represents the total species richness for native and extant species only. The original map (Fig. S4B) has a spatial resolution of ~10 km^2. To obtain mammal richness at 50 km^2 resolution, we aggregated the original raster such that each output cell contains the mean of the input cells that are encompassed by the extent of that cell.

Climate

We used the global climate regions as presented in (*55*) and available at https://rmgsc.cr.usgs.gov/outgoing/ecosystems/Global/; accessed 21/07/2020). The original dataset integrates global temperature and moisture domains and has a spatial resolution of ~250 m^2 (Fig. S4 B). Temperature domains are based on WorldClim version 2 and moisture domains are based on the aridity index (mean annual precipitation/potential evapotranspiration). See (*55*) for details. The following climate regions were represented in our dataset: Warm Temperate Dry, Tropical Dry, Sub-Tropical Dry, Cool Temperate Dry, Warm Temperate Moist, Tropical Moist, Sub-Tropical Moist, Cool Temperate Moist. We considered two versions of this dataset: the original dataset (Fig. S4 B, and Table S5), and a modified version, in which climate regions are defined by temperature regime only (Cold Temperate, Warm Temperate, Tropical and Subtropical, Fig. 4 A; Table 1; Table S6). Finally, to obtain climate data at 10 and 50 km^2 resolution, respectively, and considering that this is a categorical variable, we assigned the value corresponding to the class with the highest area percentage contained within each 10 or 50 km^2 grid cell.

All maps were re-projected to the WGS 1984 World Mercator projection.

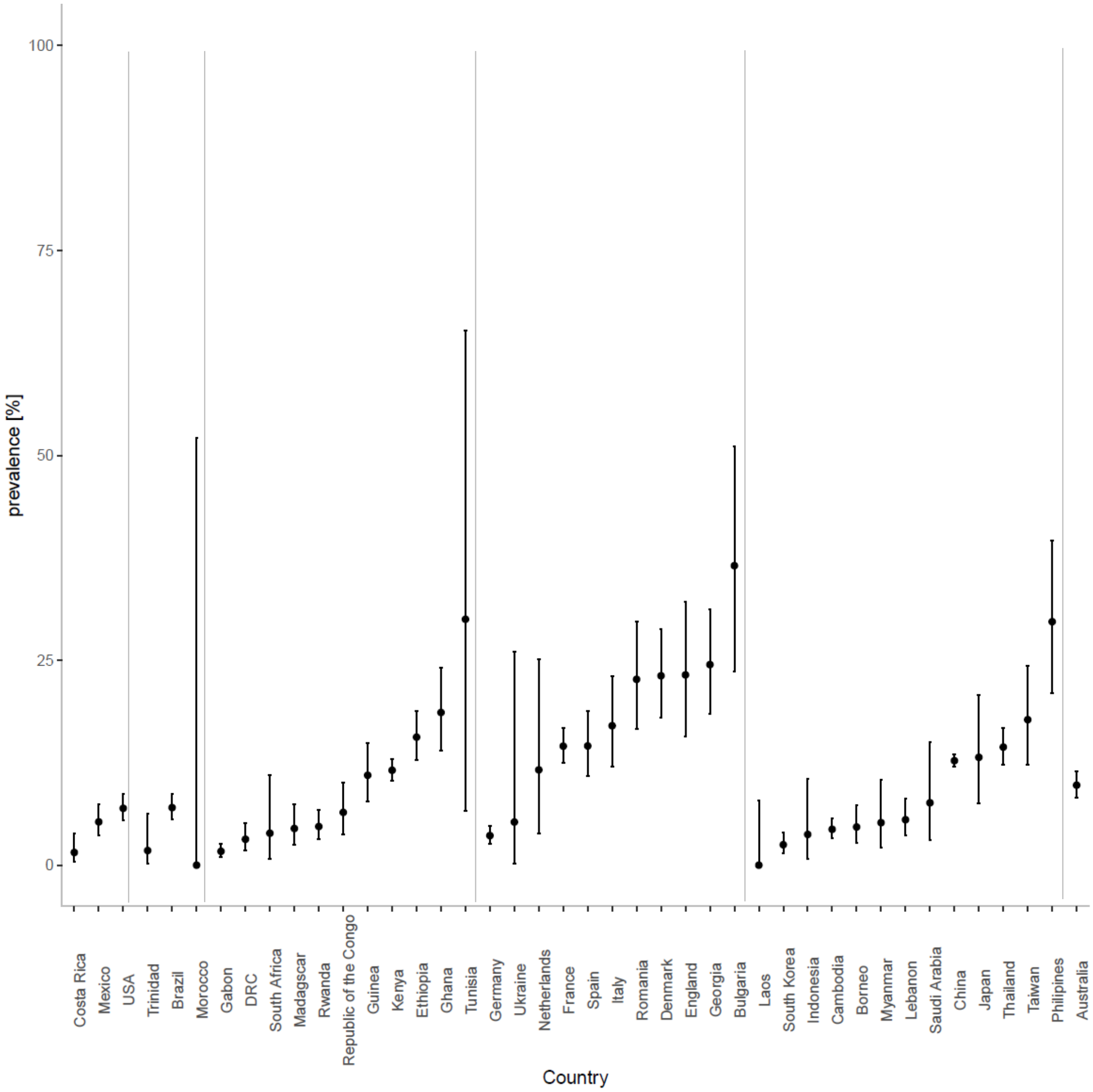

Fig. S1. Spatial variation in observed coronavirus prevalence. Shown are the mean and 95% CI for observed prevalence, calculated as the number of cases per 100 individuals and summarized at the country level using the epiR package in R v.4.0.3. Countries are ordered by ascending prevalence within continents, the latter following the order (from left to right): North America; South America, Africa, Europe, Asia, Australia.

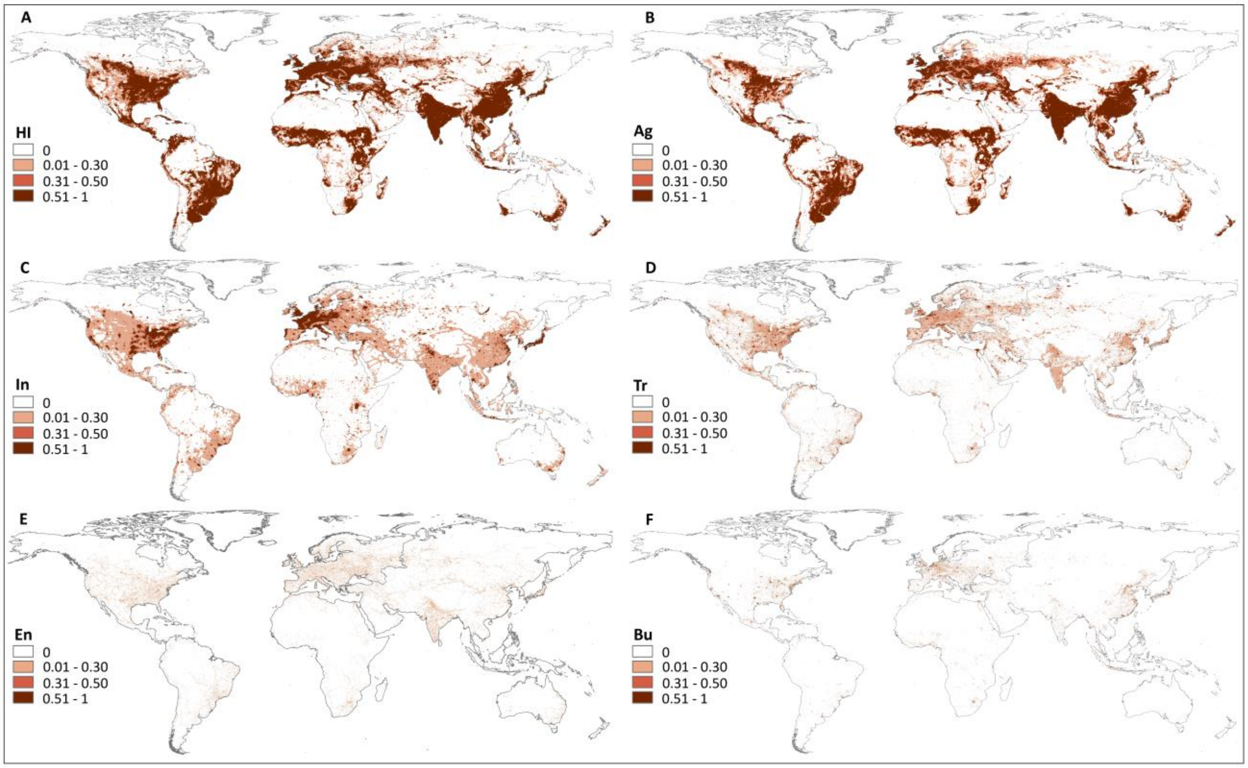

Fig. S2. Variables representing the humans stressors used in the model. The index HI (A) aggregates 14 different stressors that have been grouped into five categories of impact (B-F). For more details see (*15*).

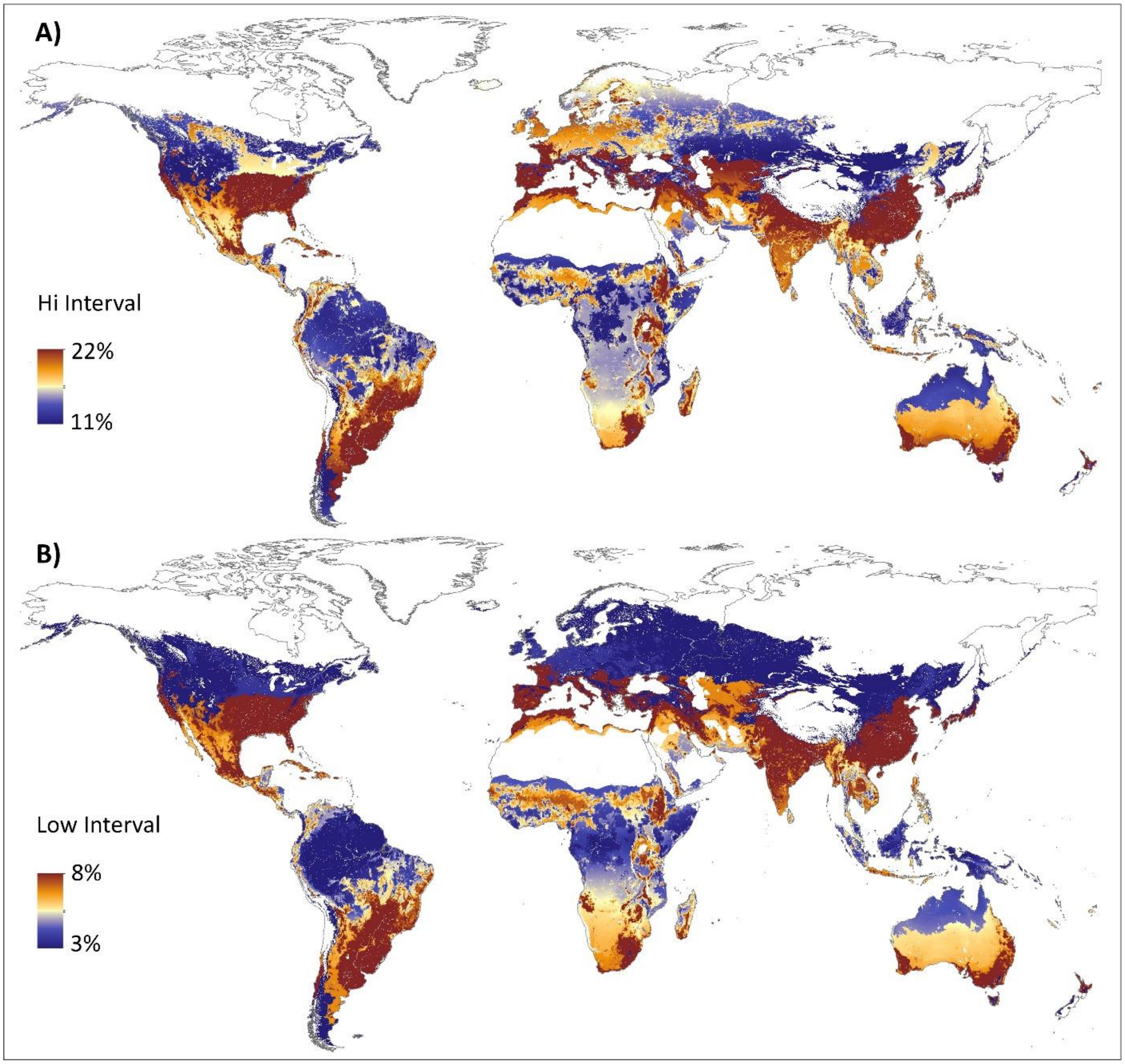

Fig S3. 95% confidence for predicted prevalence. A) Upper range (~2.5% Quantile). B) lower range (~97.5% Quantile).

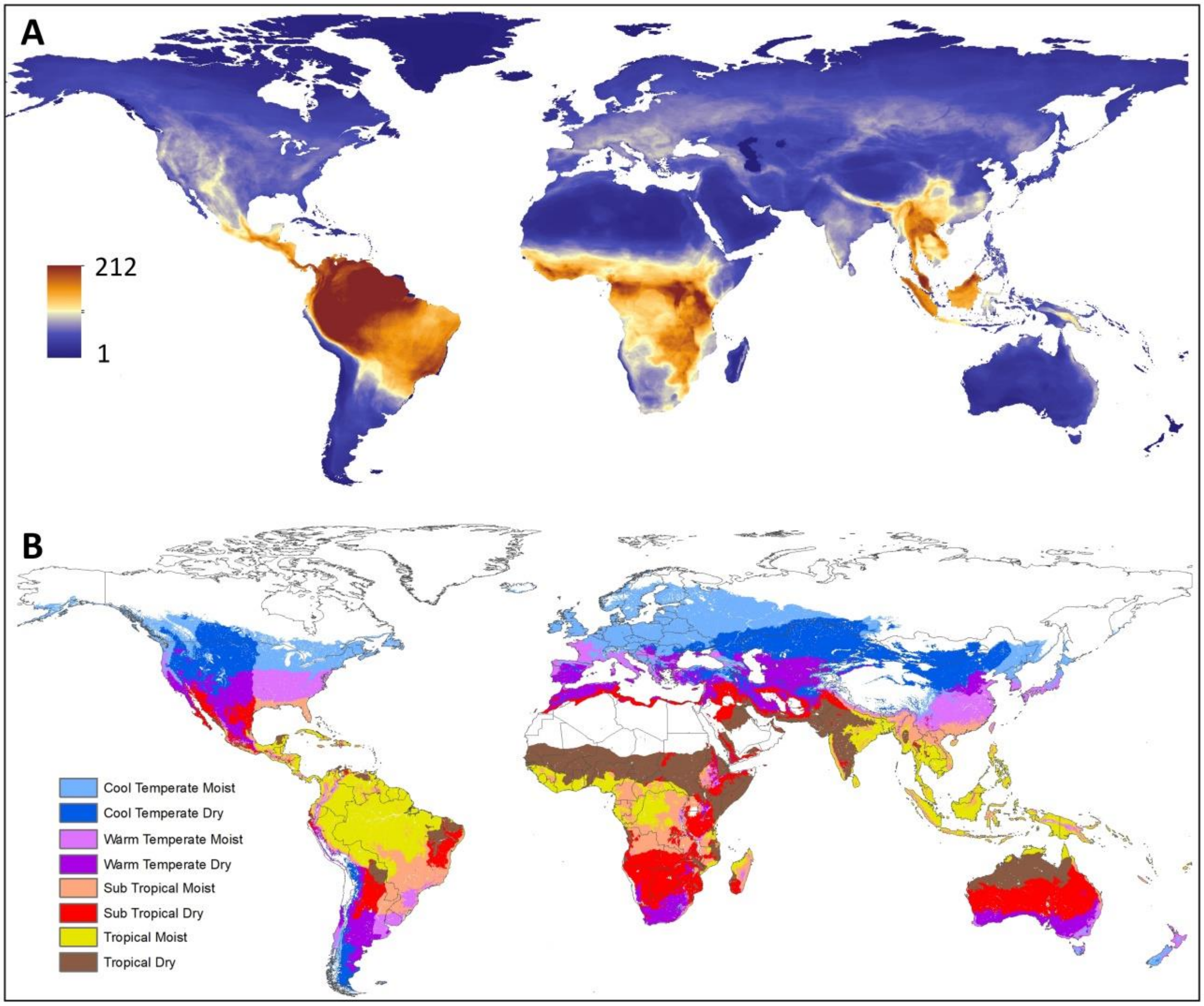

Fig. S4. Environmental variables used in the model. **A)** Mammalian species richness. **B)** World Climate Regions represented in the dataset. Climate regions not represented in the data: Boreal Moist/Dry, Polar Moist/Dry, and Desert Moist/Dry.

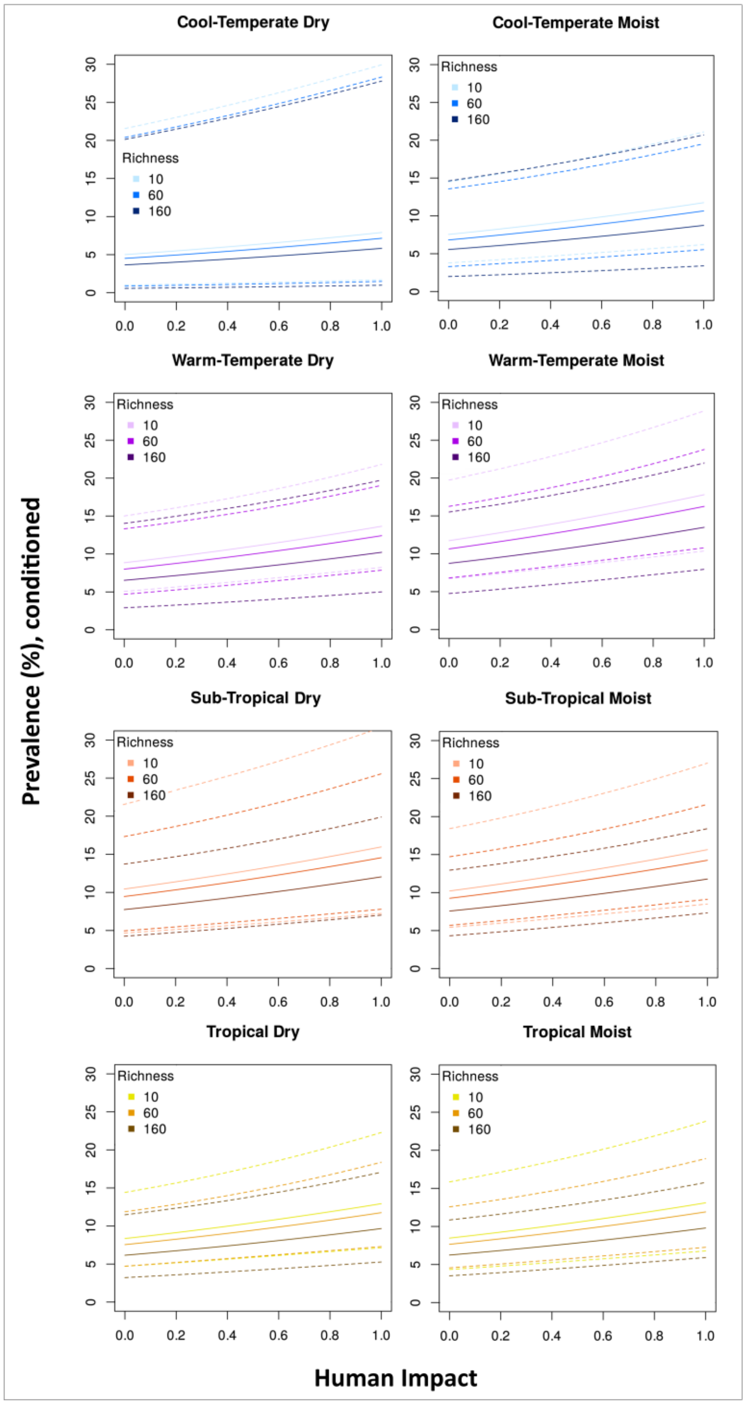

Fig. S5. Predicted relationship between human impact and coronavirus prevalence [%] for average bat species in the climate regions as defined in (*55*), and for low (10; light colours), intermediate (60; intermediate colours), and high (160; dark colours) mammal richness. Dashed lines show 95 % confidence ranges. Note that predicted prevalence is conditioned on virus presence.

Conditional model fixed effects	estimate	p-value (Wald test)
Intercept	-2.634	1.41e-06
H50	0.613	0.0002
Richness	-0.002	0.55
abs(LatDD)	0.006	0.60

Conditional model random effects	standard deviation
Climate	0.40
Species	0.97
Study	1.05

Zero-inflation model fixed effects	estimate	p-value (Wald test)
Intercept	-0.751	0.46
H50	-0.025	0.96
Richness	-0.001	0.85
abs(LatDD)	-0.005	0.78

Zero-inflation random effects	standard deviation
Climate	0.38
Species	0.61
Genus	0.36
Study	1.21

Table S3. Model coefficients for the zero-inflated binomial logistic-regression GLMM. Shown are the results for human impact at 50 km spatial resolution (H50). Note that for the zero-inflation models, larger values increase the probability of absence of infection.

**Family**	**Species**	**Prevalence Rank (high to low)**	**Sarbecovirus host***
Vespertilionidae	*Scotophilus kuhlii*	1	
Rhinolophidae	*Rhinolophus monoceros*	2	yes
Hipposideridae	*Hipposideros cervinus*	3	
Rhinolophidae	*Rhinolophus rex*	4	yes
Vespertilionidae	*Myotis davidii*	5	
Pteropodidae	*Eonycteris spelaea*	6	
Pteropodidae	*Ptenochirus jagori*	7	
Vespertilionidae	*Scotophilus heathii*	8	
Vespertilionidae	*Murina leucogaster*	9	
Vespertilionidae	*Pipistrellus coromandra*	10	
Miniopteridae	*Miniopterus pusillus*	11	
Pteropodidae	*Dobsonia moluccensis*	12	
Vespertilionidae	*Myotis siligorensis*	13	
Pteropodidae	*Cynopterus sphinx*	14	
Rhinolophidae	*Rhinolophus cornutus*	15	yes
Vespertilionidae	*Vespertilio sinensis*	16	
Pteropodidae	*Rousettus leschenaultii*	17	
Vespertilionidae	*Tylonycteris pachypus*	18	
Rhinolophidae	*Rhinolophus macrotis*	19	yes
Miniopteridae	*Miniopterus fuliginosus*	20	
Megadermatidae	*Lyroderma lyra*	21	
Hipposideridae	*Hipposideros pomona*	22	yes
Vespertilionidae	*Myotis horsfieldii*	23	
Vespertilionidae	*Myotis pilosus*	24	
Rhinolophidae	*Rhinolophus pearsonii*	25	yes
Rhinolophidae	*Rhinolophus affinis*	26	yes
Hipposideridae	*Hipposideros larvatus*	27	yes
Pteropodidae	*Pteropus lylei*	28	
Vespertilionidae	*Pipistrellus abramus*	29	
Vespertilionidae	*Myotis fimbriatus*	30	
Hipposideridae	*Hipposideros lylei*	31	
Vespertilionidae	*Myotis ikonnikovi*	32	
Rhinolophidae	*Rhinolophus coelophyllus*	33	
Emballonuridae	*Taphozous melanopogon*	34	
Pteropodidae	*Rousettus amplexicaudatus*	35	
Rhinolophidae	*Rhinolophus sinicus*	36	yes
Megadermatidae	*Megaderma spasma*	37	
Vespertilionidae	*Kerivoula papillosa*	38	
Vespertilionidae	*Myotis formosus*	39	
Rhinolophidae	*Rhinolophus shameli*	40	yes

Rhinolophidae	*Rhinolophus luctus*	41	yes
Hipposideridae	*Hipposideros swinhoei*	42	
Rhinolophidae	*Rhinolophus trifoliatus*	43	
Molossidae	*Mops plicatus*	44	
Rhinolophidae	*Rhinolophus sedulus*	45	
Pteropodidae	*Cynopterus brachyotis*	46	
Rhinolophidae	*Rhinolophus borneensis*	47	
Hipposideridae	*Hipposideros dyacorum*	48	
Vespertilionidae	*Myotis chinensis*	49	
Vespertilionidae	*Myotis macrodactylus*	50	
Hipposideridae	*Hipposideros armiger*	51	yes
Vespertilionidae	*Kerivoula hardwickii*	52	
Vespertilionidae	*Pipistrellus tenuis*	53	
Vespertilionidae	*Ia io*	54	
Vespertilionidae	*Tylonycteris robustula*	55	
Vespertilionidae	*Myotis ricketti*	56	
Rhinolophidae	*Rhinolophus malayanus*	57	yes
Vespertilionidae	*Kerivoula intermedia*	58	
Miniopteridae	*Miniopterus magnater*	59	
Pteropodidae	*Pteropus vampyrus*	60	
Hipposideridae	*Hipposideros lekaguli*	61	
Emballonuridae	*Taphozous longimanus*	62	
Vespertilionidae	*Myotis pequinius*	63	
Rhinolophidae	*Rhinolophus pusillus*	64	yes

Table S4. Predicted coronavirus prevalence for bat species whose ranges are restricted to South and South East Asia and for which a minimum of 10 individuals were sampled in the original study. *as reported in (*28*).

**Conditional model fixed effects**	estimate	p-value (Wald test)
Intercept	-2.410	5.89e-07
H10	0.489	0.000345
Richness	-0.002	0.44
abs(LatDD)	0.001	0.91

**Conditional model random effects**	standard deviation
Climate	0.328
Species	0.976
Study	1.051

**Zero-inflation model fixed effects**	estimate	p-value (Wald test)
Intercept	-0.683	0.481
Richness	-0.002	0.777
abs(LatDD)	-0.008	0.693

**Zero-inflation random effects**	standard deviation
Climate	0.381
Species	0.611
Genus	0.337
Study	1.207

Table S5. Model coefficients for the zero-inflated binomial logistic-regression GLMM. Shown are the results for climate data in which major climate regions are further sub-divided into 'dry' and 'moist'. Note that for the zero-inflation models, larger values increase the probability of absence of infection.

Original **Classification**	**classification**
Warm Temperate Moist	Warm Temperate
Warm Temperate Dry	
Tropical Moist	Tropical
Tropical Dry	
Sub Tropical Moist	Sub Tropical
Sub Tropical Dry	
Cool Temperate Moist	Cool Temperate
Cool Temperate Dry	
Warm Temperate Desert	No Data
Tropical Desert	
Sub Tropical Desert	
Cool Temperate Desert	
Polar Moist	
Polar Dry	
Polar Desert	
Boreal Moist	
Boreal Dry	
Boreal Desert	

Table S6. Original climatic regions and their classification for results presented in the main manuscript.

Table S1. List of all primary studies included in the full-text review and reason(s) for exclusion where eligibility criteria were not met.

Table S2. Coronavirus infection presence/absence in worldwide bats, and potential confounding variables included in the fitted model: Mammal_Richness - mammal species richness at the sampled locality; Climate_Region - climate region at the sampled locality. H10 - human impact at the sampled locality,10 km^2 resolution; H50 - human impact at the sampled locality, 15 km^2 resolution; Collection_Year - year in which sample was collected (if provided); Collection_Period_Start/Collection_Period_Stop - year in which first/last sample was collected; N_sampled - number of individuals sampled; N_positive - number of individuals positive for coronavirus RNA.
